# Correlation of Breast Density Grade on Mammogram With Diagnosed Breast Cancer: A Retrospective Cross-Sectional Study

**DOI:** 10.7759/cureus.27028

**Published:** 2022-07-19

**Authors:** Barka Sajjad, Nifasat Farooqi, Bushra Rehman, Ibtissam B Khalid, Namra Urooj, Saad Sajjad, Anam Mumtaz, Talha Tariq, Amina Iqbal khan, Muhammad Asad Parvaiz

**Affiliations:** 1 Breast Surgery/Surgical Oncology, Shaukat Khanum Memorial Cancer Hospital and Research Centre, Lahore, PAK; 2 Breast Surgery, Shaukat Khanum Memorial Cancer Hospital and Research Centre, Lahore, PAK; 3 Surgical Oncology, Shaukat Khanum Memorial Cancer Hospital and Research Centre, Lahore, PAK; 4 Oncology, Shaukat Khanum Memorial Cancer Hospital and Research Centre, Lahore, PAK; 5 Plastic and Reconstructive Surgery, Burn and Plastic Surgery Center, Hayatabad Medical Complex (HMC), Peshawar, PAK

**Keywords:** clinical characteristics, correlation, molecular subtypes, breast cancer, mammographic density

## Abstract

Objective

The objective of this study is to determine any association between mammographic density (MD) and breast cancer in Pakistani population. The study will also investigate relationship between mammographic breast density, clinical characteristics, and molecular tumor markers of the disease.

Methods

A retrospective review of data was carried out from January 2020 to December 2020 with stage 0-3 patients with histologically proven breast cancer included in the study. Mammograms were reviewed and density grade was recorded in accordance with "Breast Imaging Reporting and Data System (BIRADS)" guidelines. Patient age, tumor, and receptor characteristics were studied and their association with mammographic density was investigated by using chi-square test. P-value ≤0.05 was considered statistically significant.

Results

A total of 361 patients were included with a mean age of 46 years. The frequencies of BIRAD categories were as follows: category A: 8.9%, category B: 43.2%, category C: 33.5%, and category D: 14.4%. Cumulative frequency of categories B and C was 76.7%. There is a statistically significant p-value ≤0.05 association observed between age, estrogen receptors (ER) status, and T-stage versus MD. Also, majority of our patients were in T-stage category 2 or 3, which can easily be picked on mammogram.

Conclusion

Most of the breast cancer patients in our population had a mammographic density of B or C, indicating that breast cancer is more common in dense breasts. Strong significant association of mammographic density with age, ER status, and tumor stage was found in our population. Future studies need to address and confirm MD and its association with subtypes and aggressiveness of breast cancer.

## Introduction

Breast cancer is the most common cause of women morbidity and cancer-related mortality across the world [1]. In Asia, Pakistan has the highest breast cancer rate with approximately 90,000 cases being diagnosed every year with more than 40,000 deaths [2]. Breast cancer is multifactorial disease and one of its well-established and major risk factor is mammographic dense breast tissue [3]. Mammographic density (MD) refers to the percentage of dense tissue associated with stromal and epithelial proliferation of an entire breast. The common tool used for assessing MD is the breast imaging reporting and data systems (BIRADS) [4]. Women with 75% dense breast tissue have been consistently reported to be at a four to six fold higher risk of developing breast cancer than are women of similar age with little or no dense breast tissue [5,6]. One-third of all breast cancers have been found to be diagnosed in women with more than 50% density [7].

The MD distribution and prevalence of tumor subtypes have been shown to vary by race/ethnicity [8-11]; however, very limited information is available about the relationship between MD and clinical features of breast tumors in Asian populations. Asian women are known to have a higher proportion of denser breasts [10,12,13], that’s why they are diagnosed at an earlier age with breast cancer, and have a higher proportion of human epidermal growth factor receptor 2 (HER2) positive tumors compared to Western women [9,14].

The objective of the current study is to assess the relationship between MD and molecular tumor markers and clinical characteristics among women with breast cancer in Pakistan, where the breast cancer incidence rate, prevalence of established risk factors, screening practices, and MD are thought to be markedly different from those of Western women.

This article was previously presented as an abstract at the 2022 American Society of Breast Surgeons (ASBrS) annual meeting (The American Society of Breast Surgeons Official Proceedings, Volume XXIII 2022 Annual Meeting Scientific Session Abstracts. Ann Surg Oncol. 2022, 29:1-330. DOI: 10.1245/s10434-022-11703-0).

## Materials and methods

After institutional review board approval, we retrospectively reviewed 361 diagnosed and treated patients at Shaukat Khanum Memorial Cancer and Research Center from January 2020 to December 2020. Stage 0-3 patients with histologically proven breast cancer were included in the study. Patients with incomplete data regarding tumor receptor status or diagnostic mammograms were excluded from the study. All the diagnostic mammograms were reviewed by trained radiologists using the Breast Imaging Reporting and Data System (BIRADS) guidelines recommended by the American College of Radiology (fifth edition).

Clinical characteristics including the tumor size, nodal status, tumor grade, and immune histochemical markers were extracted from pathology reports. Patient age, tumor, and receptor characteristics were studied and their correlation with mammographic density was investigated using chi-square test (p<0.05). SPSS version 23 (Armonk, NY: IBM Corp.) was used for data analysis. The Institutional Review Board (IRB) of Shaukat Khanum Memorial Cancer Hospital and Research Cancer (SKMCH&RC) issued approval under IRB approval number EX-05-08-21-01.

## Results

A total of 361 patients were included in the study with the age of <40 years, 40-60 years, and >60 years. The frequencies of BIRAD categories were as follows: category A (almost entirely fat) 8.9%, category B (scattered fibro-glandular densities) 43.2%, category C (heterogeneously dense) 33.5%, and category D (extremely dense) 14.4%. Cumulative frequency of categories B and C was 76.7% (Table 1).

**Table 1:** Bifurcation of demographic and clinical characteristics of breast cancer patients with respect to mammographic density. ER: estrogen receptors; PR: progesterone receptors; HER2/neu: human epidermal growth factor receptor 2

Variables	Characteristics	Mammographic density				
		A	B	C	D	p-Value
Age (years)	<40	2 (6.2%)	33 (21.2%)	48 (39.7%)	24 (46.2%)	0.001
	40-60	21 (65.6%)	103 (66%)	63 (52.1%)	24 (46.2%)	
	Above 60	9 (28.1%)	20 (12.8%)	10 (8.3%)	4 (7.7%)	
ER status	Negative	7 (21.9%)	40 (25.6%)	50 (41.3%)	12 (23.1%)	0.01
	Positive	25 (78.1%)	116 (74.4%)	71 (58.7%)	40 (76.9%)	
PR status	Negative	14 (43.8%)	80 (51.3%)	73 (60.3%)	21 (40.4%)	0.06
	Positive	18 (56.2%)	76 (48.7%)	48 (39.7%)	31 (59.6%)	
HER2/neu	Negative	25 (78.1%)	113 (72.4%)	81 (66.9%)	37 (71.2%)	0.592
	Positive	7 (21.9%)	43 (27.6%)	40 (33.1%)	15 (28.8%)	
T-stage	T1	8 (25.0%)	24 (15.4%)	12 (9.9%)	7 (13.5%)	0.01
	T2	20 (62.5%)	88 (56.4%)	65 (53.7%)	21 (40.4%)	
	T3	4 (12.5%)	44 (28.2%)	44 (36.4%)	24 (46.2%)	
Grade	I	-	2 (1.3%)	4 (3.3%)	2 (3.8%)	0.09
	II	20 (62.5%)	87 (55.8%)	51 (54.5%)	31 (59.6%)	
	III	12 (37.5%)	67 (42.9%)	66 (54.5%)	19 (36.5%)	

**Figure 1:**
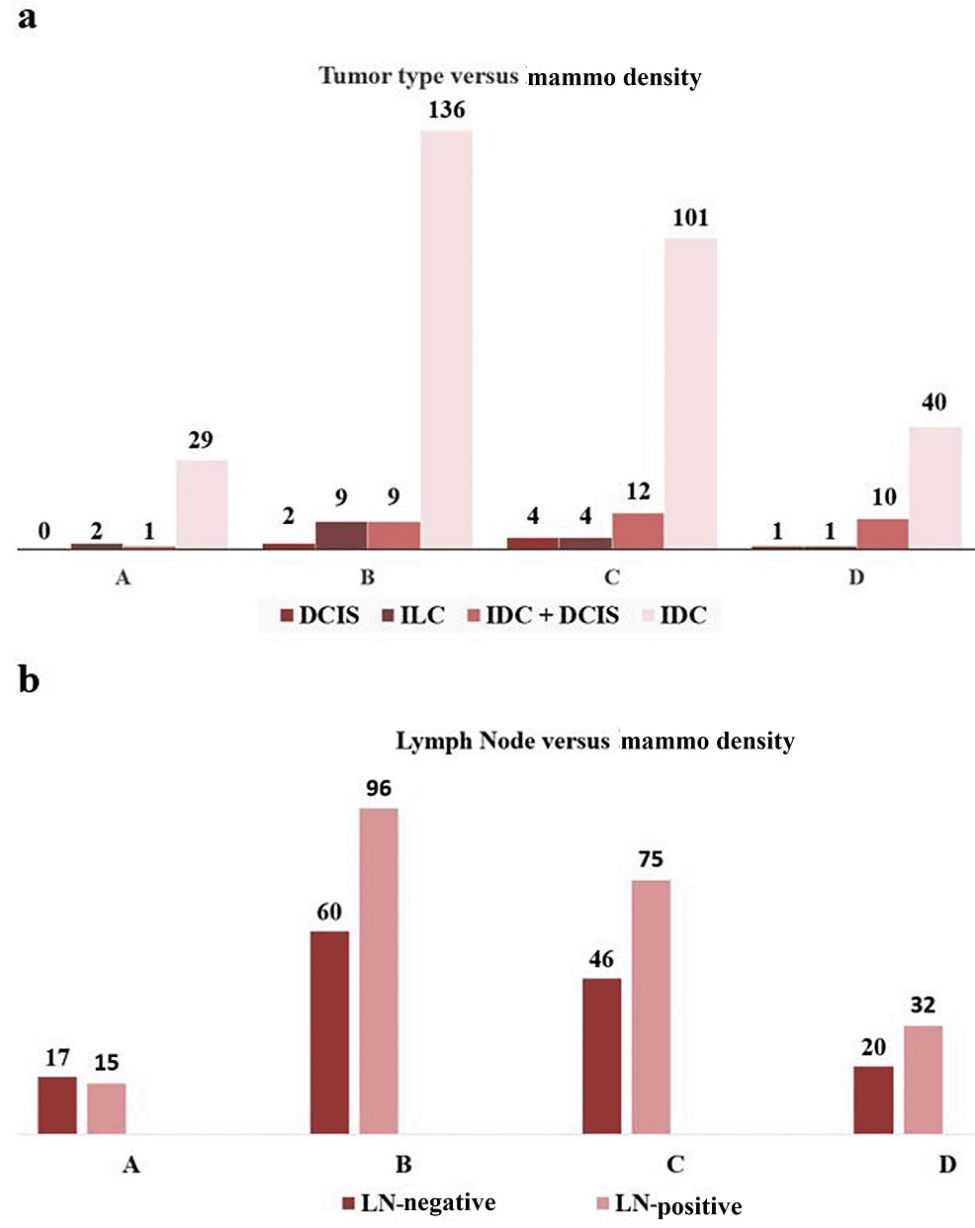
The image shows (a) mammographic density versus tumor type and (b) mammographic density versus lymph node. DCIS: ductal carcinoma+ductal carcinoma in situ; ILC: invasive lobular carcinoma; IDC: invasive ductal carcinoma; LN: lymph node

Majority of patients had invasive ductal carcinoma (IDC) 84.8%, invasive lobular carcinoma (ILC) 16%, and invasive ductal carcinoma+ductal carcinoma in situ (DCIS) and DCIS alone were 8.9% and 1.9%, respectively (Figure 1a). Grade II was 52.4%, estrogen receptors (ER), progesterone receptors (PR), and human epidermal growth factor receptor 2 (HER 2/neu) positivity were found to be 69.8%, 47.9%, and 25.8% respectively. Most patients were T2 (tumor <5 cm) 53.7%, followed by T3 (tumor >5 cm) 32.1%, and T1 (tumor <2 cm) 13.9% (Table 1). Lymph node-positive patients were 60.4%; fine needle aspiration cytology (FNA) as shown in Figure 1b.

There is a statistical significant p-value ≤0.05 association observed between age, ER status, and T-stage versus mammographic density (Table 1). PR status and grade of tumor showed marginal statistical significance versus mammographic density (Table 1). Also, majority of our patients were in T-stage category 2 or 3, which can easily be picked on mammogram.

## Discussion

Mammographic density is a well-established risk factor; however, the current evidence regarding mammographic density and breast cancer association according to different tumor characteristics is unclear and at variance [15-18]. Few previous studies report no clear difference in association by tumor characteristics [16,18,19], while others are at the opinion that relation between mammographic density and breast cancer risk differs by ER status [20,21], invasiveness [20,22], and tumor size [17,22].

Most [17-25] but not all [22] prior studies have reported a stronger relation of mammographic density with large tumors versus small tumors, which could reflect delays in diagnosis due to reduced sensitivity of mammography and/or aggressive tumor biology [6].

According to our results, patients above 40 years of age with mammographic density of category B have three fold increased risk of breast cancer as compared with the same category in less than 40 years of age patients. Majority of patients in our population with diagnosed breast cancers had a mammographic density of B and C proving that breast cancer is more common in dense breasts. So, we report a strong association between mammographic density categories B and C with breast cancer.

Studies by Ding et al. and Conroy et al. found an association between increased MD and ER+ tumors, whereas a study by Yaghjyan et al. found an association between increased MD and ER- tumors [21,26-28]. In addition, a study by Sartor et al. found an association between increased MD and ER- tumors though only among clinically detected cancers instead of those identified on screening [29,30]. Our study shows that there is a strong significant association between mammographic density and estrogen receptor status.

A smaller case-control study done in Korean population did not observe association between MD and breast cancer risk by tumor markers defined subtypes [31]. In contrast, a Chinese study reported association between high MD and the HER2 enriched tumor subtype [32]. Interestingly, no association was found between mammographic density and HER2 tumors in our population.

There are several studies that show positive association between larger tumor size and higher MD, we identify positive association between mammographic density and histologic grade II of breast tumors [29,33-36]. However, other studies have reported mammographic density to be either positively [36] or negatively associated with histologic grade [34,37]. A strong association between mammographic density and grade was assumed to reflect a biological relationship between a high amount of breast glandular tissue and a low degree of tumor differentiation (or high histologic grade) [36]. Mostly grade II tumors with invasive components had MD B and C. The MD categories B and C have positive association with lymph node positivity.

The limitation of this study is that it’s a retrospective study with small sample size, deficient demographic data like parity, menopausal status, and the lack of standardization of BIRADS category on mammograms. The strength of this study is it’s unique of its kind that only diagnostic mammograms were used with very limited data available on the subject in our population.

## Conclusions

Most of the breast cancer patients in our population had a mammographic density of B or C, indicating that breast cancer is more common in dense breasts. Strong significant association of mammographic density with age, ER status, and tumor stage was found in our population. However, further studies with larger sample size need to be done with more comprehensive information on breast cancer risk factors. Mammographic density and its association with subtypes and aggressiveness of breast cancer. Radiologists should be extra vigilant in categories B and C to make sure they don’t miss cancer. As majority of our patients presented in T2/late stage, we conclude that mammography should be supplemented with other imaging modalities to pick small cancers on screening mammograms. Mammographic density can accurately predict breast cancer.
